# Inhibition of cyclooxygenase‐2 enhanced intestinal epithelial homeostasis via suppressing β‐catenin signalling pathway in experimental liver fibrosis

**DOI:** 10.1111/jcmm.16730

**Published:** 2021-06-19

**Authors:** Linhao Zhang, Yang Tai, Chong Zhao, Xiao Ma, Shihang Tang, Huan Tong, Chengwei Tang, Jinhang Gao

**Affiliations:** ^1^ Laboratory of Gastroenterology & Hepatology State Key Laboratory of Biotherapy West China Hospital Sichuan University Chengdu China; ^2^ Department of Gastroenterology West China Hospital Sichuan University Chengdu China

**Keywords:** celecoxib, COX‐2, intestinal homeostasis, liver fibrosis, β‐catenin

## Abstract

The intestinal barrier dysfunction is crucial for the development of liver fibrosis but can be disturbed by intestinal chronic inflammation characterized with cyclooxygenase‐2 (COX‐2) expression. This study focused on the unknown mechanism by which COX‐2 regulates intestinal epithelial homeostasis in liver fibrosis. The animal models of liver fibrosis induced with TAA were established in rats and in intestinal epithelial–specific COX‐2 knockout mice. The impacts of COX‐2 on intestinal epithelial homeostasis via suppressing β‐catenin signalling pathway were verified pharmacologically and genetically in vivo. A similar assumption was tested in Ls174T cells with goblet cell phenotype in vitro. Firstly, disruption of intestinal epithelial homeostasis in cirrhotic rats was ameliorated by celecoxib, a selective COX‐2 inhibitor. Then, β‐catenin signalling pathway in cirrhotic rats was associated with the activation of COX‐2. Furthermore, intestinal epithelial–specific COX‐2 knockout could suppress β‐catenin signalling pathway and restore the disruption of ileal epithelial homeostasis in cirrhotic mice. Moreover, the effect of COX‐2/PGE2 was dependent on the β‐catenin signalling pathway in Ls174T cells. Therefore, inhibition of COX‐2 may enhance intestinal epithelial homeostasis via suppression of the β‐catenin signalling pathway in liver fibrosis.

## 1 INTRODUCTION

Intestinal epithelial homeostasis, which depends on the balance of continuous proliferation, differentiation and cell death of intestinal epithelia, is the cornerstone of the barrier function of intestinal epithelia.^1^, ^2^, ^3^, ^4^ It has been known that dysfunction of the intestinal epithelial barrier is involved in the pathogenesis of chronic liver diseases.^5^, ^6^ Recent data have showed that intestinal epithelial homeostasis was disrupted because of an imbalance between the proliferation of intestinal epithelial cells and the differentiation of goblet cells in rodents with liver fibrosis.^7^, ^8^ A reduction in the number of well‐differentiated goblet cells apparently results in the lower output of mucus and intestinal barrier dysfunction.^9^ It would be easy for noxious substances in the intestinal lumen to pass through its defective barrier and then enter into hepatic sinusoid damaging the liver.^10^, ^11^ Interception of this process may be helpful to slow down the progression of cirrhosis. However, the underlying mechanism of intestinal epithelial dyshomeostasis during liver fibrosis remains largely unknown.

Expression of cyclooxygenase‐2 (COX‐2) induced by various inflammatory events in intestinal mucosa has showed detrimental impacts on liver fibrosis via its catalytic products, for example prostaglandin E2 (PGE2).^12^, ^13^, ^14^, ^15^, ^16^ Our previous study ever showed that celecoxib, a selective COX‐2 inhibitor, could improve the integrity of the intestinal barrier in cirrhotic rats partly through increasing expressions of epithelial tight junction protein.^12^ However, it has not yet been elucidated the COX‐2‐involved mechanism by which intestinal epithelial dyshomeostasis happens.

It has been reported that β‐catenin signalling pathway plays a crucial role in the regulation of intestinal epithelial proliferation and differentiation.^17^, ^18^ An inactive β‐catenin molecule in the intestinal epithelia is phosphorylated and hydrolysed by a proteolytic degradation complex containing glycogen synthase kinase (GSK) 3β.^19^ During inflammation, GSK3β can be phosphorylated (pGSK3β) and inactivated.^20^ Therefore, β‐catenin is no longer degraded (non‐phospho β‐catenin) but functions as a transcriptional factor. The target genes of β‐catenin, such as c‐MYC and cyclin D1, will be up‐regulated to stimulate the proliferation of intestinal epithelia.^21^, ^22^, ^23^ It is not clear whether β‐catenin signalling pathway is related to COX‐2 expression during inflammatory stimulation of liver fibrosis.

This study was aimed to verify the roles of COX‐2 on intestinal epithelial dyshomeostasis in vivo with pharmacological and genetical way and to elucidate its underlying mechanisms related to β‐catenin signalling pathway under the circumstance of liver fibrosis. The positive results would provide useful therapeutic target for cirrhotic patients.

## 2 MATERIALS AND METHODS

### 2.1 Animal models of liver fibrosis

The animal experiments were approved by the Ethics Committees of Sichuan University and were conducted according to the regulations of Sichuan University. Thirty‐six male *Sprague Dawley* rats weighing 200‐250 g (aged 7‐8 weeks) were obtained from the Experimental Animal Center of Sichuan University (Chengdu, China). Rats were allocated to the control group, TAA (Sigma‐Aldrich) group and TAA+C group with 12 in each. The control group received peritoneal injection of normal saline. Liver fibrosis was induced in the TAA group by peritoneal injection of TAA for 16 weeks (200 mg/kg/3 d for the initial 8 weeks and 100mg/kg/3 d for the following 8 weeks). In the TAA+C group, peritoneal injection of TAA was the same as the TAA group, except for the supplementation of celecoxib (20 mg/kg/d) by gavage starting from the 8th week of TAA injection in the TAA+C group.

Intestinal epithelial–specific knockout of COX‐2 mouse line (C57/B6 background) was derived by using Cre‐loxP, with villin promoter driving Cre expression (villin‐Cre^+^). COX‐2^fl/fl^ and villin‐Cre^+^ mice were crossed in our facilities. Male COX‐2^fl/fl^/villin‐Cre^+^ mice (V‐COX‐2^KO^) aged 7‐8 weeks and their age‐matched littermate controls COX‐2^fl/fl^/villin‐Cre^‐^ mice (V‐COX‐2^fl/fl^) were used. There were 4 mice in each group. Liver fibrosis was induced in mice by peritoneal injection of TAA for 8 weeks (200 mg/kg/3 d).

### 2.2 Cell culture and treatments

Human colon cancer cell line Ls174T was obtained from the American Type Culture Collection (ATCC) and verified by STR profiling. Ls174T was known to express mucus in vitro.^7^ Ls174T cells were maintained in Dulbecco's modified Eagle's medium (DMEM, HyClone) containing 10% foetal bovine serum (FBS, Biological Industries, Cromwell), 100 U/mL penicillin and 100 U/mL streptomycin (HyClone). Cells were routinely incubated with 5% CO_2_ at 37°C.

Celecoxib and Wnt‐C59 were obtained from TopScience Limited Liability Company, PGE2 from Selleck and LiCl from Sigma‐Aldrich Corp. For celecoxib, PGE2 and Wnt‐C59 treatments, drugs were dissolved in dimethyl sulfoxide (DMSO) before adding to the culture medium (the final concentration of DMSO was 0.1%). For LiCl treatment, it was dissolved in a serum‐free culture medium before adding to the culture medium. After the indicated treatment was added for 24 hours of incubation, cells were harvested for further experimentation.

### 2.3 Assay of alanine aminotransferase (ALT) and aspartate aminotransferase (AST)

Blood from the portal vein was collected from animals. Then, blood was left standing for about 30 minutes. Serum was obtained by centrifugation at 1000 *g* for 15 minutes. Serum ALT and AST were analysed by West China‐Frontier Pharma by using Cobas 6000 c501 (Roche).

### 2.4 Enzyme‐linked Immunosorbent Assay (ELISA)

Concentrations of lipopolysaccharide (LPS) in serum, and PGE2 and MUC2 of ileum and Ls174T cells were determined by ELISA kits according to the instruction (Cloud‐Clone Corp).

### 2.5 Western blot analysis

The whole proteins from frozen ilea or cells were extracted on ice by using RIPA buffer (Beyotime Biotechnology) with addition of phosphatase inhibitor cocktail and protease inhibitor cocktails (Bimake). Proteins of 50 μg from tissues or 30 μg from cells were resolved by 10% SDS‐PAGE for further experiments, as described before.^7^ Primary antibodies included CDK4 (Proteintec), P21 (SAB, College Park), COX‐2 (Abcam), c‐MYC (Abcam), cyclin D1 (Huabio), PCNA (Huabio), cleaved‐caspase 3 (CST, Danvers), active β‐catenin (CST), total β‐catenin (CST), total GSK3β (CST), phospho‐GSK3β (CST) and GAPDH (ABclonal).

### 2.6 Cell counting kit‐8 (CCK8) assay

The cells were seeded in a 96‐well plate to the density of 50%‐70% confluence. CCK8 assay was performed according to the instruction (Dojindo). The optical density was then measured by a Thermo microplate reader (Thermo Fisher Scientific) at 450 nm.

### 2.7 Histopathological evaluation

Livers and ilea from animals were fixed by 4% polyformaldehyde, embedded by paraffin and sectioned (thickness of 5 μm). Staining was performed with haematoxylin and eosin (H&E), Sirius red or periodic acid–Schiff (PAS). For Sirius red staining, Ishak's fibrosis stage of the liver (with the Ishak scale ranges from 0 = no fibrosis to 6 = cirrhosis) was evaluated. For PAS staining, the ileal sections were analysed. Villi length, perimeters of villi, goblet cell number per perimeter of villi and theca area of goblet cell were measured by ImagePro Plus 6.0 (Media Cybernetics). These methods were described previously.^7^, ^24^ For PAS staining of Ls174T cells, cells were plated on glass chamber slides. Cells were fixed by Carnoy's fluid (ratio of ethanol to acetic acid, 3:1) for 15 minutes. PAS staining was then performed according to the instructions (Solarbio).

### 2.8 Immunohistochemistry (IHC) staining

The ileal sections were routinely deparaffinized. Antigen retrieval was performed in sodium citrate buffer (10 mmol/L, Ph = 6.0) with a pressure cooker. After blocking with 3% hydrogen peroxide, and incubation with 10% goat serum, subsequent primary antibodies were applied. Primary antibodies included Ki‐67 (Abcam), COX‐2 (Abcam), cleaved‐caspase 3 (CST), cyclin D1 (Huabio), total GSK3β (CST) and phospho‐GSK3β (CST). After incubation overnight at 4°C, a goat anti‐rabbit secondary antibody (Abcam) was used. Finally, staining with diaminobenzoate (ZSGB‐BIO) was performed before counterstaining with haematoxylin. Negative control was done by adding PBS instead of primary antibody. The images were captured by an optical microscope (CX41, Olympus) equipped with a camera (DP72, Olympus). The AOD was measured by ImagePro Plus 6.0 (Media Cybernetics) as described before.^7^

### 2.9 Immunofluorescent (IF) staining

For IF of tissues, PFA‐fixed and paraffin‐embedded ileal sections were used. The ileal sections were routinely deparaffinized. Antigen retrieval was performed in sodium citrate buffer (10 mmol/L, Ph = 6.0) with a pressure cooker. After permeabilization with 0.2% Triton X‐100 for 15 minutes, the sections were incubated with 10% goat serum for 40 minutes and subsequent primary antibodies overnight at 4°C. Primary antibodies included active β‐catenin (CST) and E‐cadherin (CST). Then, further incubation was done with the corresponding fluorescent secondary antibody at 37°C for 1 hour. Sections were counterstained with DAPI before visualization. The images of sections were captured by an optical microscope (CX41, Olympus) equipped with a camera (DP72, Olympus).

For IF of cells, the cells were plated on glass chamber slides. Treatment was the same as mentioned above. Fixation was performed with 4% PFA for 15 minutes. Permeabilization and incubation with antibodies were the same as the aforementioned methods for animal tissues. Negative control was done by adding PBS instead of primary antibody in all assays. Primary antibodies included active β‐catenin (CST) and Ki‐67 (Abcam). The images were captured by Axio Imager Z2.

### 2.10 Flow cytometry for cell apoptosis

After cells were collected, Annexin V Apoptosis Detection Kit with propidium iodide (PI, Beijing 4A Biotech Co) was used according to the manufacturer's instruction. Cytoflex (Beckman Coulter) was used for detection.

### 2.11 Statistical analysis

All experiments consisted of a minimum of 3 replicates. Quantitative variables are shown as mean ± SD. Student's *t* test and one‐way analysis of variance (followed by a Tukey's post hoc analysis) were utilized, where applicable. GraphPad Prism 5 software (San Diego) was applied for analysis. *P* lower than .05 was deemed statistically significant.

## 3 RESULTS

### 3.1 Disruption of intestinal epithelial homeostasis in cirrhotic rats was ameliorated by celecoxib

**FIGURE 1:**
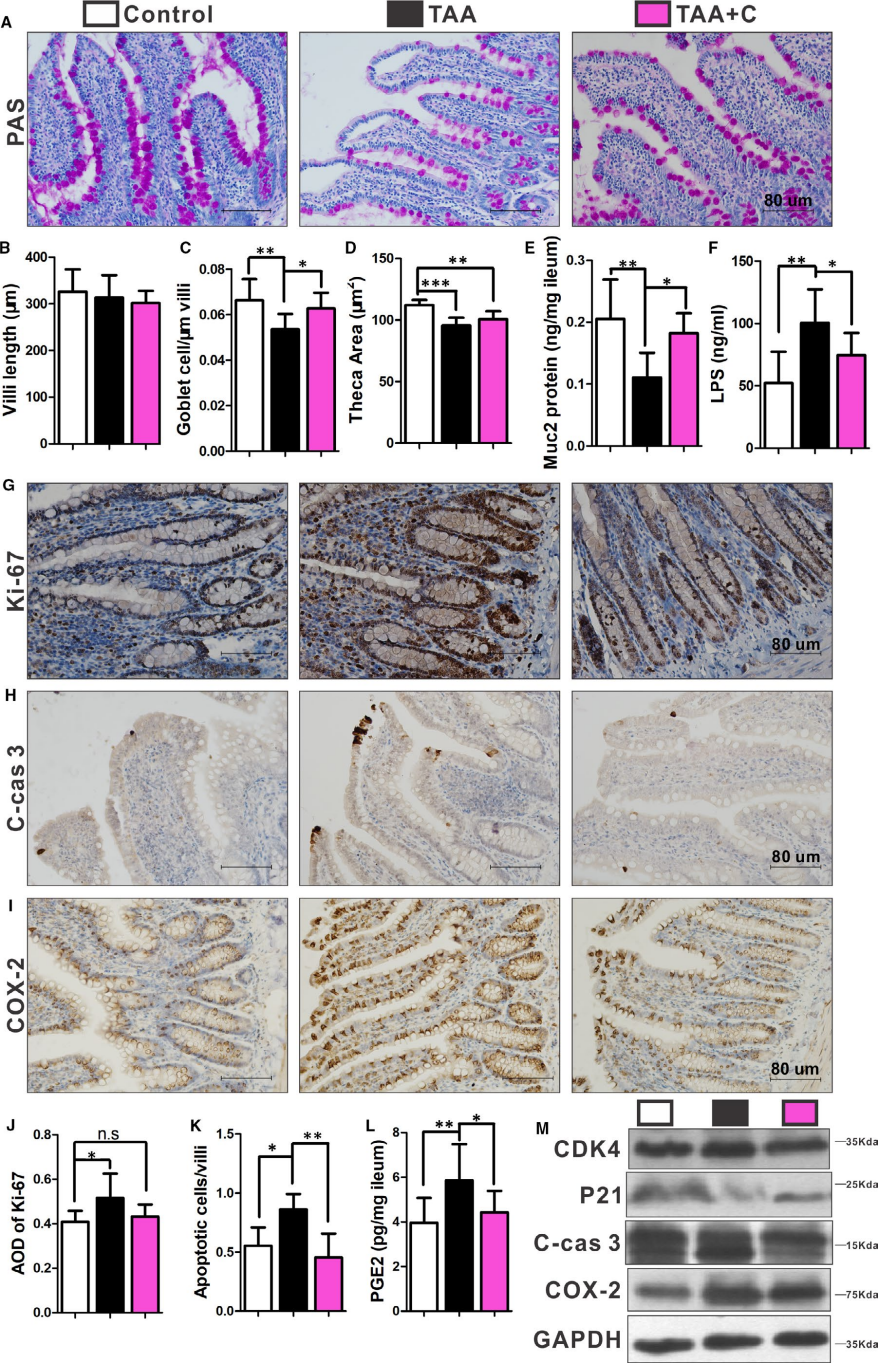
Disruption of intestinal epithelial homeostasis in cirrhotic rats was ameliorated by celecoxib. Periodic acid–Schiff (PAS) staining showed reduced goblet cells in the TAA group, while in the TAA+C group, the goblet cells seemed to be more in the TAA+C group compared with that in the TAA group (A). Villi length was comparable among the 3 groups (B; n = 12 in each group). Goblet cell number/μm of villi and theca area of goblet cells in the TAA group were lowest, which was partly reversed by celecoxib (C and D; n = 7‐8 in each group for theca area of goblet cells; n = 10‐12 in each group for goblet cell count). The mucus component Muc2 protein was significantly lower in the TAA group, which was increased by celecoxib treatment (E, n = 7‐10 in each group). Serum lipopolysaccharide (LPS) in portal vein was significantly higher in the TAA group, and it was reduced in the TAA+C group (F, n = 8‐11 in each group). An increase in Ki‐67‐positive (G), cleaved‐caspase 3–positive (H) and COX‐2‐positive (I) ileal epithelia was observed in the TAA group. AOD of Ki‐67 and number of cleaved‐caspase 3–positive epithelia were significantly increased only in the ileum of the TAA group (J‐K; n = 7‐9 in each group for Ki‐67; n = 5‐6 in each group for cleaved‐caspase 3). Prostaglandin 2 (PGE2) was also significantly higher in the TAA group (L, n = 9‐12 in each group). Protein expressions of CDK4, cleaved‐caspase 3 and COX‐2 were higher, and the level of P21 was lower in the TAA group (M, n = 7‐10 in each group). All experiments consisted of a minimum of 3 replicates. Data are shown as mean ± SD; *P < .05, **P < .01, ***P < .001; one‐way ANOVA with Tukey's post hoc test. C‐cas 3, cleaved‐caspase 3

Liver fibrosis induced by TAA was showed with either gross or Sirius red staining. It was significantly alleviated by celecoxib treatment (Figure S1A,C). The increased serous ALT in TAA group (*P <* .05) was potentially lower by celecoxib treatment but was still significantly higher than that in the control group (Figure S1D). There was no difference in ileal villi length among three groups (Figure 1A,B; Figure S1B). However, the theca of goblet cells in ileum of the TAA group was significantly smaller and less compared with that of the control rats (*P <* .05; Figure 1C,D). Also, the expression of Muc2 protein in rat ilea was significantly lower in the TAA group than that in the control group (Figure 1E). The goblet cells of villi, theca area and the expression of Muc2 protein were partially improved in the TAA+C group compared with that in the TAA group (Figure 1A,C‐E). LPS level in portal circulation was higher in the TAA group compared with that in the control group (*P <* .05), which was decreased by celecoxib treatment (Figure 1F).

Ileal epithelial proliferation detected by Ki‐67 expression was significantly higher in TAA group than that in the control group (*P <* .05). But it almost fell back to the control level after celecoxib treatment (Figure 1G,J). Consistently, compared with control group, significant increase of proliferation‐related protein CDK4 associated with lower level of proliferation suppressive protein P21 was observed in the TAA group (*P <* .05; Figure 1M). These changes were greatly reversed by celecoxib treatment (*P <* .05; Figure 1M). The number of apoptotic cells showed by cleaved‐caspase 3 in TAA group was the largest among the three groups (*P <* .05; Figure 1H,K,M).

### 3.2 Activation of COX‐2 and β‐catenin signalling pathway in cirrhotic rats was suppressed by celecoxib

**FIGURE 2:**
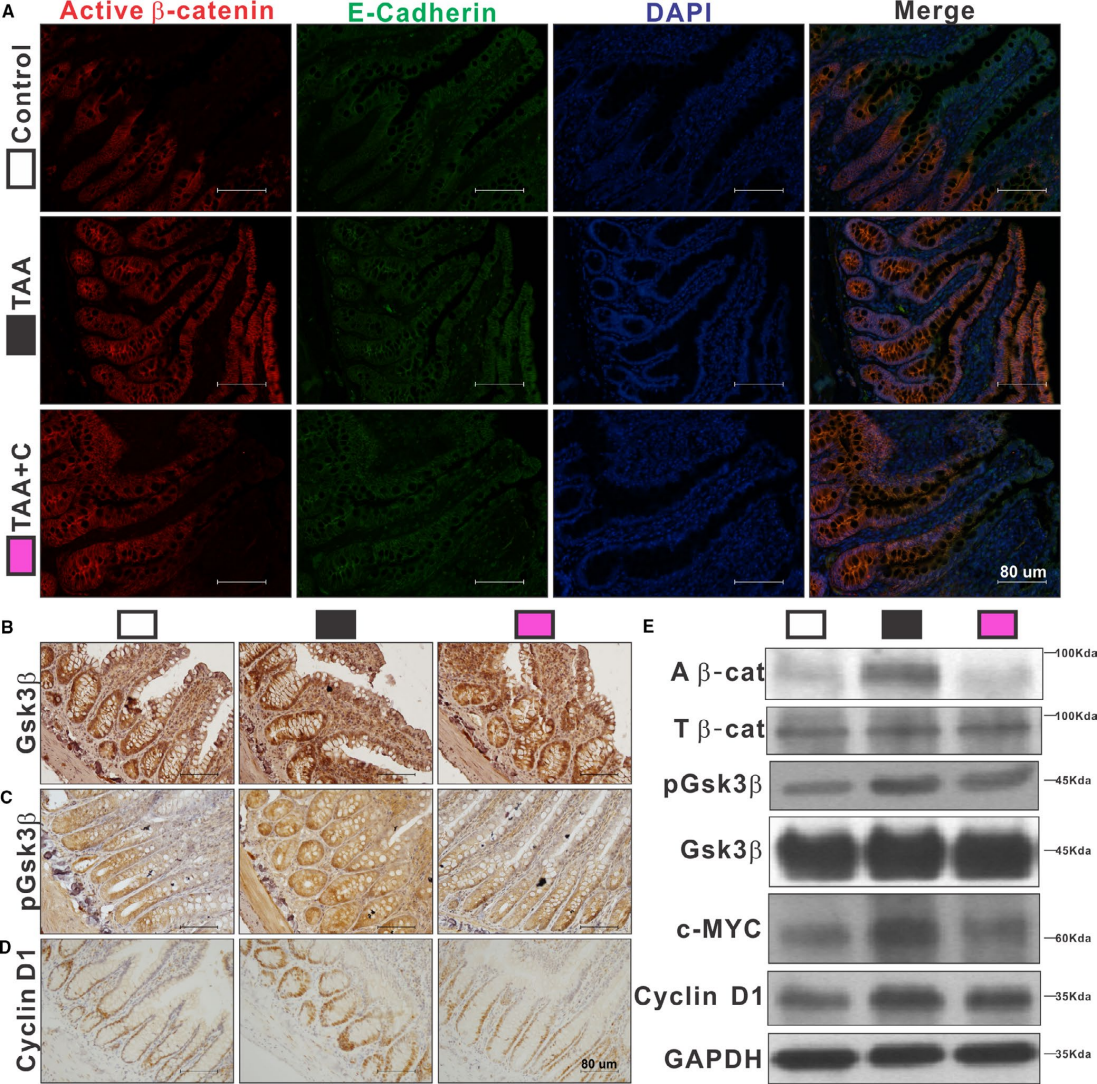
Activation of β‐catenin signalling pathway in cirrhotic rats was suppressed by celecoxib. Active β‐catenin was visualized by IF staining using E‐cadherin as background (A). IHC staining of pGSK3β (C) and cyclin D1 (D) was stronger in the TAA group, with similar GSK3β (B) among the 3 groups. Protein expressions of active β‐catenin, pGSK3β, c‐MYC and cyclin D1 were higher in the TAA group, while the level of total β‐catenin and GSK3β were similar among the 3 groups (E, n = 6‐10 in each group). All experiments consisted of a minimum of 3 replicates. Data are shown as mean ± SD; one‐way ANOVA with Tukey's post hoc test. A β‐cat, active β‐catenin; T β‐cat, total β‐catenin

As the β‐catenin signalling pathway plays a crucial role in the regulation of intestinal epithelial homeostasis, the effect of COX‐2 on the β‐catenin signalling pathway was verified. The overexpression of COX‐2 in the ileal epithelial cells of rats treated with TAA could be significantly suppressed by celecoxib (*P <* .05; Figure 1I,M). Furthermore, the increased ileal level of PGE2, a catalytic product of COX‐2, was also greatly decreased with celecoxib treatment (*P <* .05; Figure 1L). Although quantification of GSK3β, which degradates active β‐catenin, did not show significant differences among the three groups (*P >* .05), the ileal concentrations of pGSK3β (an inactive form of GSK3β) and downstream elements of β‐catenin such as cyclin D1 and c‐MYC were much higher in TAA group than in control one (*P <* .05; Figure 2B‐E). Ileal β‐catenin was greatly activated by TAA and presented as non‐phospho β‐catenin (*P <* .05; Figure 2A,E). Celecoxib effectively inhibited the β‐catenin signalling pathway mentioned above (*P <* .05; Figure 2).

### 3.3 Disruption of ileal epithelial homeostasis in cirrhotic mice was blocked by intestinal epithelial–specific COX‐2 knockout

**FIGURE 3:**
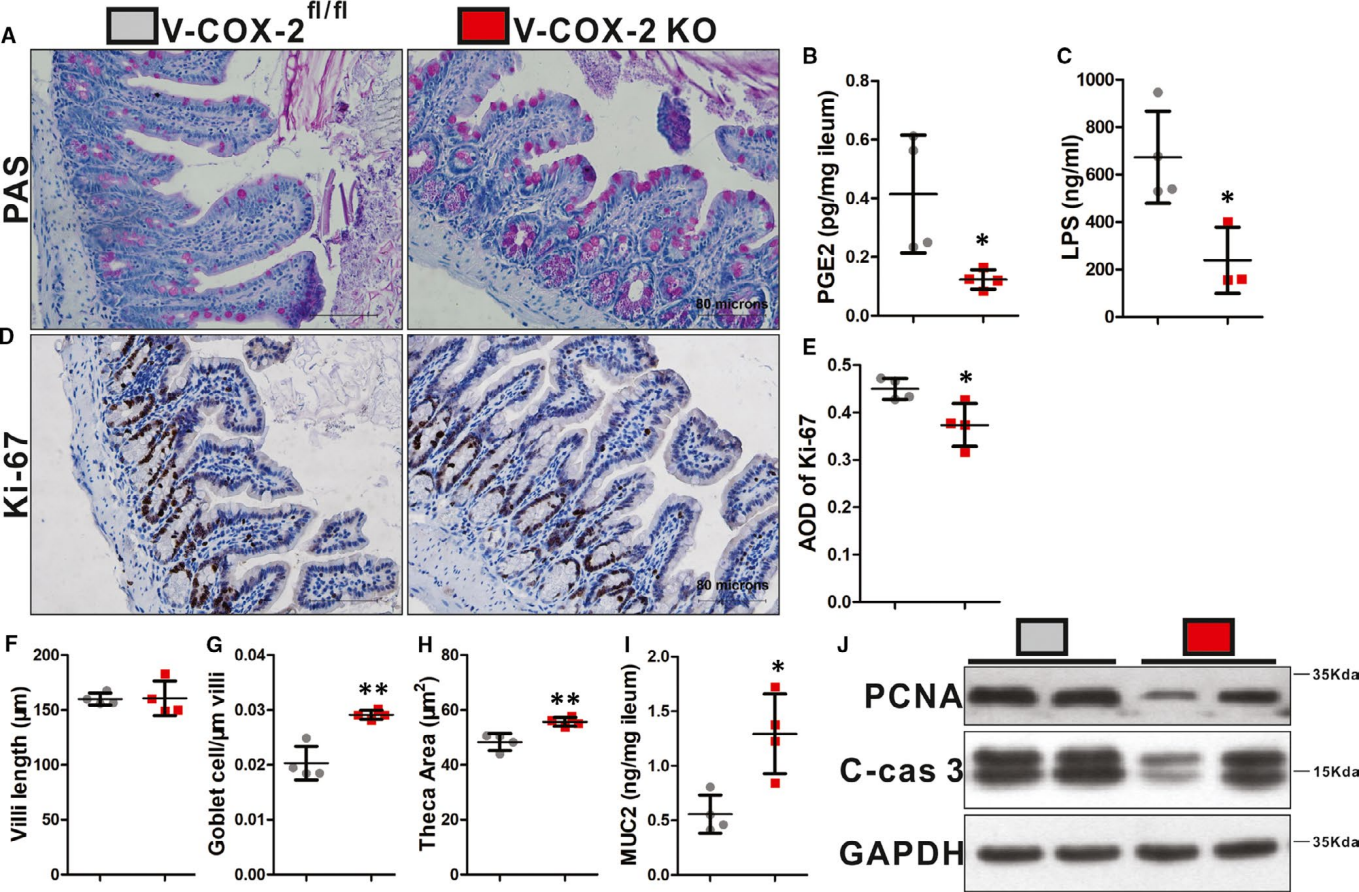
Disruption of ileal epithelial homeostasis in cirrhotic mice was restored by intestinal epithelial–specific COX‐2 knockout. PAS staining showed increased goblet cells in V‐COX‐2^KO^ mice (A). Serum LPS and ileal PGE2 were significantly lower in V‐COX‐2^KO^ mice (B‐C). V‐COX‐2^KO^ mice had less Ki‐67‐positive cells in the ileum (D). AOD of Ki‐67 was reduced in V‐COX‐2^KO^ mice (E). Villi length was similar (F). Goblet cell number/μm of villi, theca area of goblet cells and mucus component Muc2 protein in V‐COX‐2^KO^ mice were significantly increased (G‐I). Expressions of PCNA and cleaved‐caspase 3 were decreased in V‐COX‐2^KO^ mice (M). n = 3‐4 in each group in all experiments. All experiments consisted of a minimum of 3 replicates. Data are shown as mean ± SD; *P < .05, **P < .01, ***P < .001; two‐tailed *t* test. C‐cas 3, cleaved‐caspase 3. V‐COX‐2^KO^, intestinal epithelial–specific COX‐2 knockout mice. V‐COX‐2^fl/fl^, COX‐2^fl/fl^/villin‐Cre^‐^ mice

There was no significant difference of fibrostic extent in the livers between intestinal epithelial–specific COX‐2 knockout mice (V‐COX‐2^KO^) and littermate controls (V‐COX‐2^fl/fl^; Figure S2A,C), *P >* .05. However, both serum ALT and AST were significantly decreased in the V‐COX‐2^KO^ mice (*P <* .05; Figure S2D‐E). The average ileal concentration of PGE2 in V‐COX‐2^KO^ mice was lower than that in littermate controls (*P <* .05; Figure 3B). Moreover, LPS level in portal circulation of V‐COX‐2^KO^ mice was obviously lower than that of littermate controls (*P <* .05; Figure 3C). The ileal theca of goblet cells in V‐COX‐2^KO^ mice became more and larger than those in the control mice (*P* < .05; Figure 3A,G‐H), while there was no difference in villi length between groups (*P* > .05; Figure 3F, Figure S2B). Consistently, quantification of ileal MUC2 in V‐COX‐2^KO^ mice was significantly higher than that in littermate controls (*P* < .05; Figure 3I). With regard to the proliferation of ileal epithelia of V‐COX‐2^KO^ mice, the percentage of Ki‐67, PCNA and cleaved‐caspase 3 levels were greatly decreased compared to control mice (*P <* .05; Figure 3D‐E,3J). Therefore, not only pharmacologically but also genetically, COX‐2 was presented as a crucial pivot for ileal epithelial homeostasis in cirrhotic mice.

### 3.4 Inhibition of β‐catenin signalling pathway in cirrhotic mice by intestinal epithelial‐specific COX‐2 knockout

**FIGURE 4:**
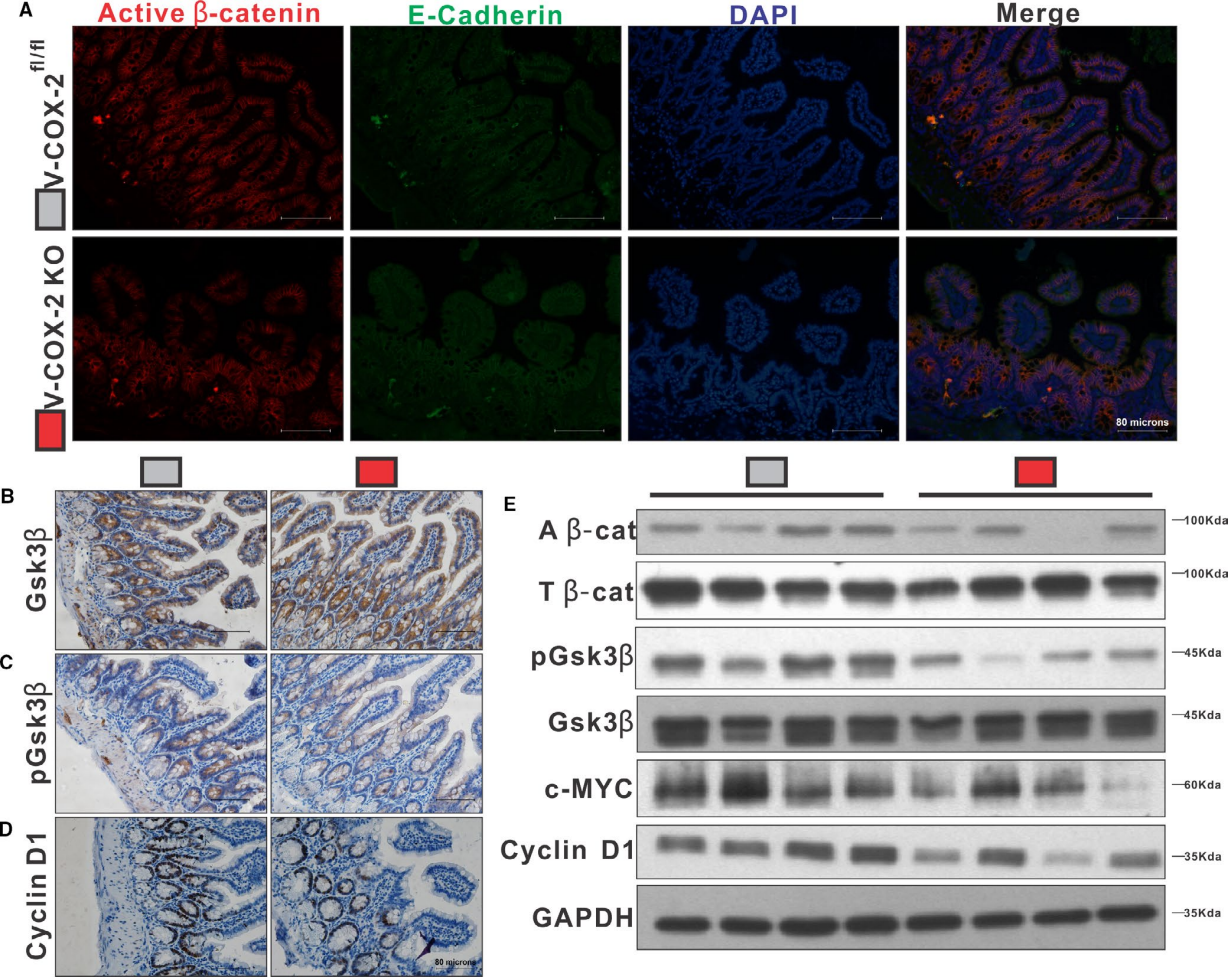
Intestinal epithelial β‐catenin activity was suppressed in intestinal epithelial–specific COX‐2 knockout mice. Active β‐catenin was visualized by IF staining using E‐cadherin as background (A). IHC intensity of pGSK3β (C) and cyclin D1 (D) was lower in V‐COX‐2^KO^ mice, but similar GSK3β was observed (B). Protein expressions of active β‐catenin, pGSK3β, c‐MYC and cyclin D1 were less in V‐COX‐2^KO^ mice, while the level of total β‐catenin and GSK3β were similar between the 2 groups (E). n = 3‐4 in each group in all experiments. All experiments consisted of a minimum of 3 replicates. Data are shown as mean ± SD; two‐tailed *t* test. A β‐cat, active β‐catenin; T β‐cat, total β‐catenin. V‐COX‐2^KO^, intestinal epithelial‐specific COX‐2 knockout mice. V‐COX‐2^fl/fl^, COX‐2^fl/fl^/villin‐Cre^‐^ mice

**FIGURE 5:**
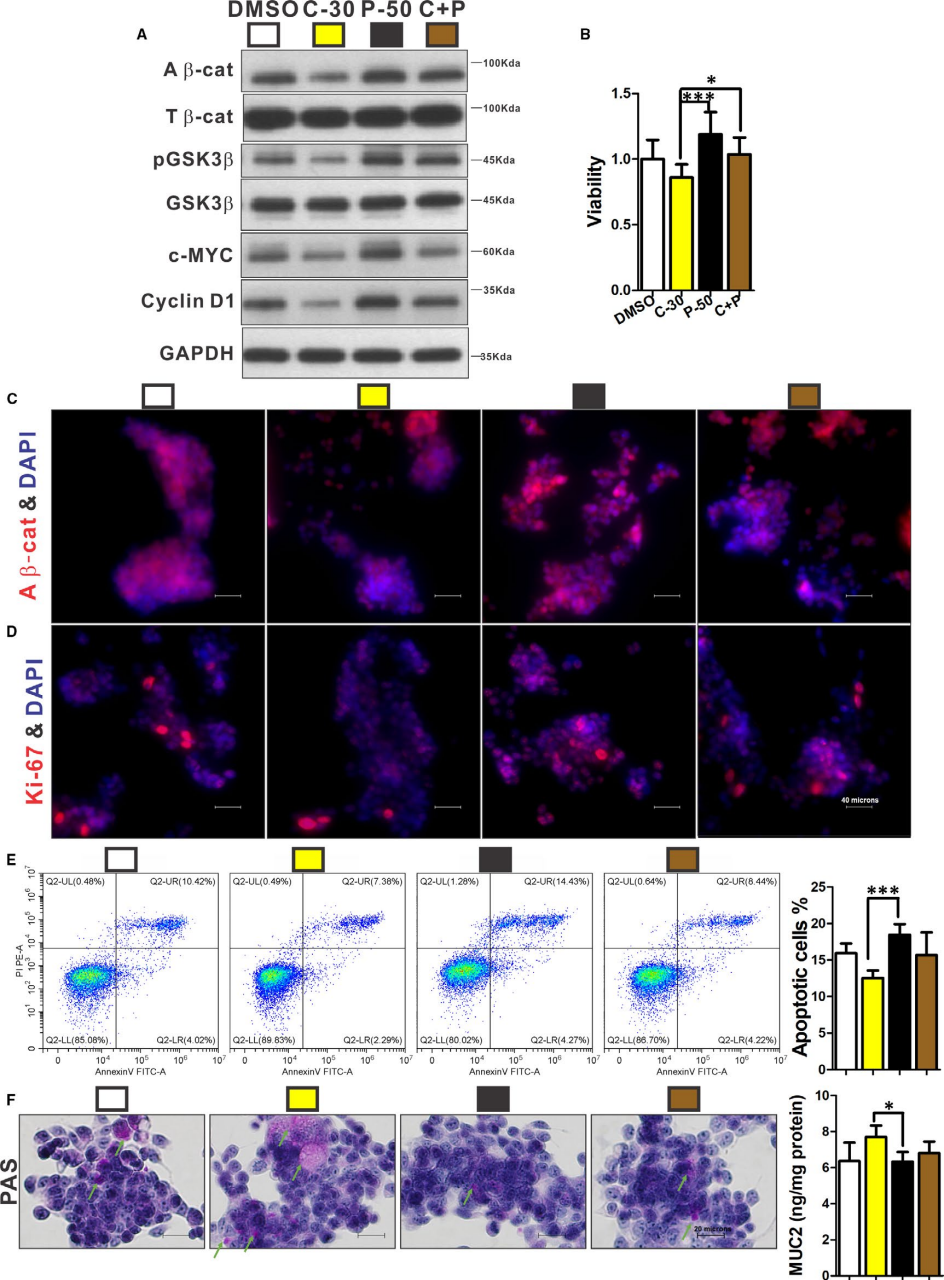
COX‐2 regulated proliferation, differentiation and apoptosis via PGE2 in Ls174T cells. Cells were treated by 30 μmol/L celecoxib alone (C‐30), 50 μmol/L PGE2 alone (P‐50) and a combination of 30 μmol/L celecoxib and 50 μmol/L PGE2 (C + P), respectively. Protein expressions of active β‐catenin, pGSK3β, c‐MYC and cyclin D1 were reduced by celecoxib and a combination of celecoxib and PGE2, while PGE2 had opposite effects (A). Cell viability could be increased by PGE2, and the effect could be reversed by the addition of celecoxib (B). Ki‐67‐positive (D) and β‐catenin‐positive (C) cells were lowered by celecoxib, PGE2 had opposite effects, and these effects could be abolished by a combination of celecoxib and PGE2. Apoptotic cells were decreased by celecoxib treatment, and PGE2 induced apoptosis, while the combination of celecoxib and PGE2 did not significantly alter apoptotic rate (E). The Muc2 expression and mucus (green arrow) were significantly induced by celecoxib compared with PGE2 (F). n ≥ 3 in each group. All experiments consisted of a minimum of 3 replicates. Data are shown as mean ± SD; *P < .05, **P < .01, ***P < .001; one‐way ANOVA with Tukey's post hoc test. A β‐cat, active β‐catenin; T β‐cat, total β‐catenin

The expression of non‐phospho β‐catenin was inhibited in ilea of V‐COX‐2^KO^ mice (*P <* .05; Figures 4A,5E). The concentrations of pGSK3β, and downstream elements of β‐catenin, cyclin D1 and c‐MYC, were also inhibited in V‐COX‐2^KO^ mice (*P <* .05), whereas the expression of GSK3β was comparable between two groups (Figure 4B‐E).

### 3.5 COX‐2 regulated proliferation, differentiation and apoptosis via PGE2 in Ls174T cells

Celecoxib was used to treat mucus‐expressing Ls174T cells. The optimal concentration of celecoxib was 30 μmol/L. It not only significantly suppressed PGE2 concentration but also inhibited the viability of Ls174T cells (*P <* .05; Figure S3). Then, cells were treated by 30 μmol/L celecoxib alone (C‐30), 50 μmol/L PGE2 alone (P‐50) and a combination of 30 μmol/L celecoxib and 50 μmol/L PGE2 (C + P), respectively. C‐30 significantly suppressed viability (Figure 5B), proliferation (Figure 5D) and apoptosis (Figure 5E) and increased mucus expression of Ls174T cells (Figure 5F). Celecoxib also suppressed the β‐catenin signalling pathway (Figure 5A,C). Contrarily, P‐50 had opposite effects on these cellular processes (*P <* .05), while C + P treatment could mitigate the effects of celecoxib (*P <* .05; Figure 5).

### 3.6 The effects of COX‐2 on Ls174T cells were mediated by the β‐catenin signalling pathway

**FIGURE 6:**
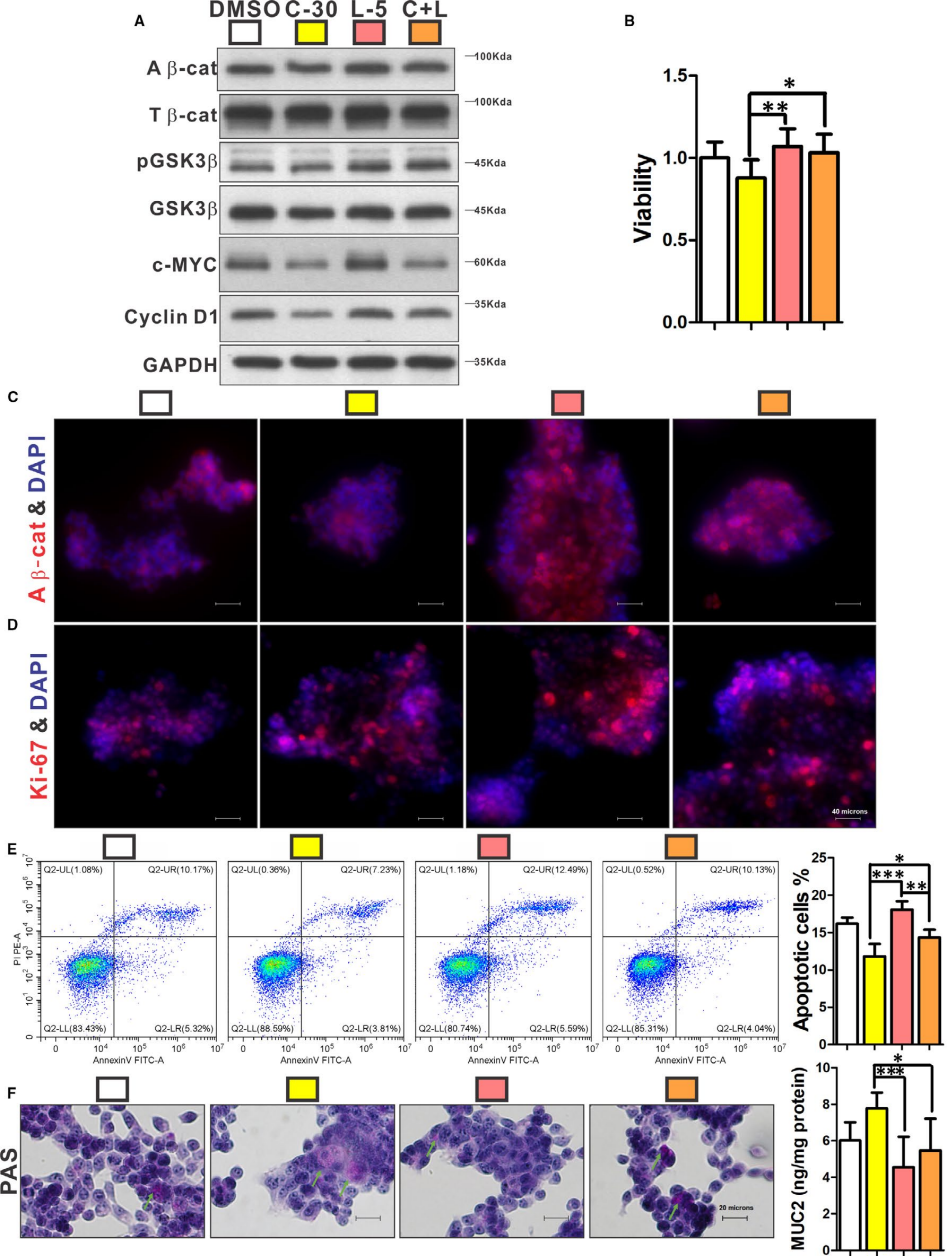
The effects of COX‐2 on Ls174T cells were mediated by the β‐catenin signalling pathway. Cells were treated by 30 μmol/L celecoxib alone, 5 mmol/L LiCl alone (L‐5) and a combination of 30 μmol/L celecoxib and 5 mmol/L LiCl (C + L), respectively. Protein expressions of active β‐catenin, pGSK3β, c‐MYC and cyclin D1 were reduced by celecoxib, while LiCl increased active β‐catenin, pGSK3β, c‐MYC and cyclin D1; and the combination of celecoxib and LiCl reversed the effects of celecoxib (A). Cell viability could be increased by LiCl, and the effect could be partly inhibited by addition of celecoxib (B). Ki‐67‐positive (D) and β‐catenin‐positive (C) cells were increased by LiCl, and these effects could be reduced partially by a combination of celecoxib and LiCl. Apoptotic cells were increased by LiCl treatment, which could be reversed by celecoxib (E). The Muc2 expression and mucus (green arrow) were significantly decreased by LiCl compared with celecoxib (F). n ≥ 3 in each group. All experiments consisted of a minimum of 3 replicates. Data are shown as mean ± SD; *P < .05, **P < .01, ***P < .001; one‐way ANOVA with Tukey's post hoc test. A β‐cat, active β‐catenin; T β‐cat, total β‐catenin

Ls174T cells were next treated by 30 μmol/L celecoxib alone, 5 Mmol/L LiCl alone (L‐5; an activator of β‐catenin signalling pathway) and a combination of 30 μmol/L celecoxib and 5 mmol/L LiCl (C + L), respectively. L‐5 significantly increased viability (Figure 6B), proliferation (Figure 6D) and apoptosis (Figure 6E) and reduced mucus expression of Ls174T cells (Figure 6F). L‐5 also augmented the β‐catenin pathway compared with celecoxib treatment (Figure 6A,C). However, C + L treatment could partially reverse the effect of LiCl (Figure 6).

### 3.7 The effects of PGE2 on Ls174T cells were mediated by the β‐catenin signalling pathway

**FIGURE 7:**
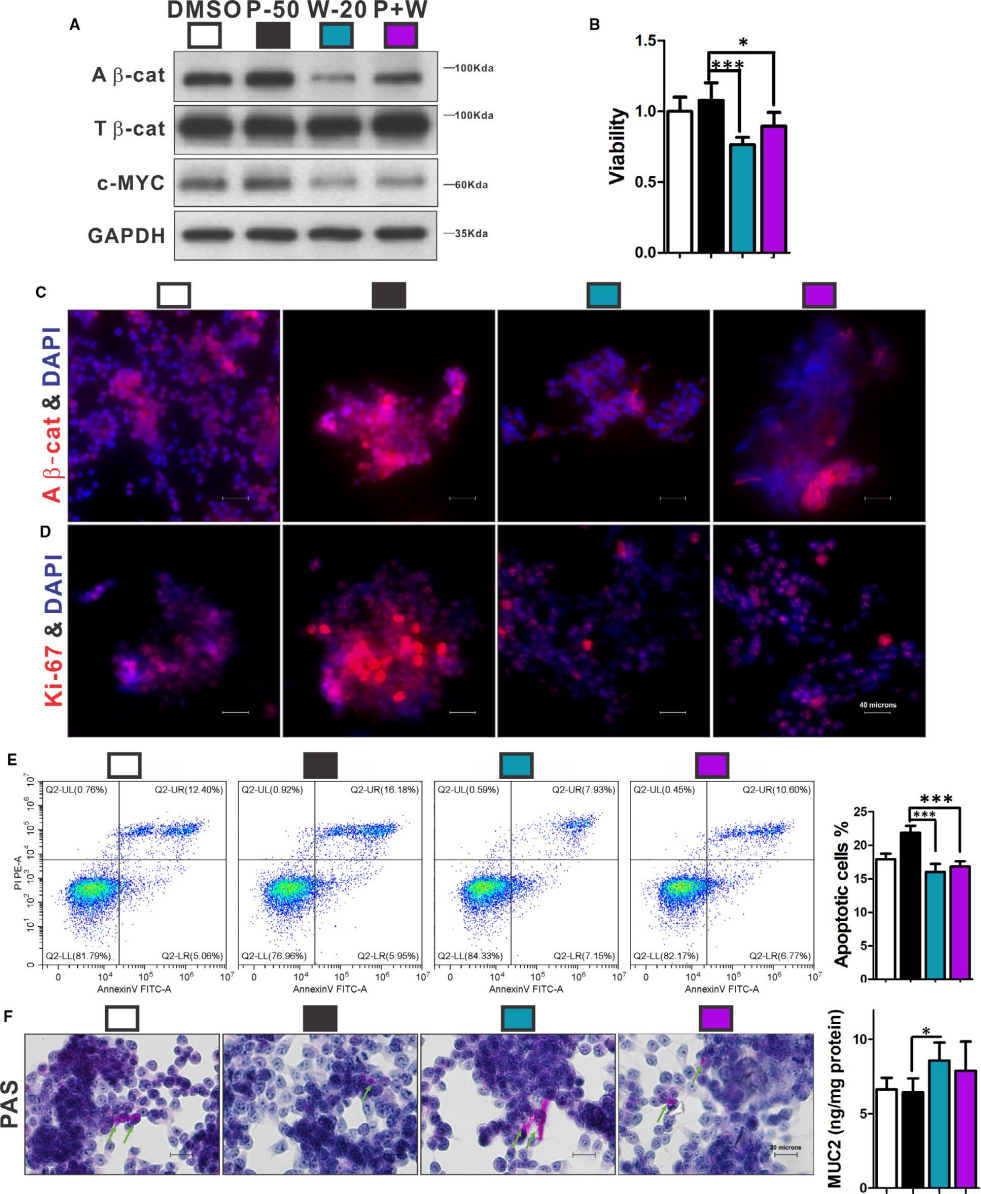
The effects of PGE2 on Ls174T cells were mediated by the β‐catenin signalling pathway. Cells were treated by 50 μmol/L PGE2 alone, 20 μmol/L Wnt‐C59 alone (W‐20) and a combination of 50 μmol/L PGE2 and 20 μmol/L Wnt‐C59 (P + W), respectively. Protein expressions of active β‐catenin and c‐MYC were reduced by Wnt‐C59, and combination of PGE2 and Wnt‐C59 suppressed c‐MYC, while PGE2 increased active β‐catenin and c‐MYC (A). Cell viability could be reduced by Wnt‐C59, and the effect could be partly inhibited by addition of PGE2 (B). Ki‐67‐positive (D) and β‐catenin‐positive (C) cells were decreased by Wnt‐C59, and these effects could be ameliorated partially by a combination of PGE2 and Wnt‐C59. Apoptotic cells were decreased by Wnt‐C59 treatment compared with PGE2 treatment (E). The Muc2 expression and mucus (green arrow) were significantly up‐regulated by Wnt‐C59 compared with PGE2 (F). n ≥ 3 in each group. All experiments consisted of a minimum of 3 replicates. Data are shown as mean ± SD; *P < .05, **P < .01, ***P < .001; one‐way ANOVA with Tukey's post hoc test. A β‐cat, active β‐catenin; T β‐cat, total β‐catenin

Ls174T cells were treated by 50 Μmol/L PGE2 alone, 20 μmol/L Wnt‐C59 alone (W‐20; a WNT inhibitor suppressing β‐catenin signalling pathway) and a combination of 50 μmol/L PGE2 and 20 μmol/L Wnt‐C59 (P + W), respectively. W‐20 significantly decreased viability (Figure 7B), proliferation (Figure 7D) and apoptosis (Figure 7E) and up‐regulated mucus expression of Ls174T cells (Figure 7F). Wnt‐C59 also suppressed the β‐catenin signalling pathway (Figure 7A,C). Nevertheless, P + W treatment could partly reverse the effect of Wnt‐C59 (*P <* .05; Figure 7).

## 4 DISCUSSION

As a physical barrier, intestinal mucus and epithelia are fundamental in the separation of detrimental substances in the gut lumen and are essential to prevent liver injury from these toxins.^25^ Epithelial proliferation, differentiation and apoptosis are very important to maintain the intestinal epithelial homeostasis.^1^, ^2^, ^3^ This study presented that the disruption of intestinal homeostasis related to the development of liver fibrosis in rats was characterized as increased proliferation and apoptosis but poor differentiation of goblet cells in ileal epithelial cells. As a result, LPS levels in the portal vein of these rats were greatly enhanced.

Mucus secreted from goblet cells covers the intestinal epithelia and is known as the first line of defence for intestinal mucosal surface.^7^ The intestinal mucus barrier is impaired due to the reduction of goblet cells in liver fibrosis.^7^, ^8^ However, little is known why goblet cells are reduced in liver fibrosis. Our data showed that the reduction of goblet cells was accompanied by the overexpression of COX‐2 in the intestinal epithelia. COX‐2 is expressed at low levels in the gut under normal physiological conditions and is highly induced in response to inflammation and intestinal injury.^12^ COX‐2 and its product PGE2 made the goblet cells poorly differentiated in this study in vivo and in vitro. Such observation was confirmed by the experiment with celecoxib and intestinal epithelial–specific COX‐2 knockout mice. Beside reduced viability, proliferation and apoptosis of intestinal epithelia, celecoxib greatly enhanced mucus output through decreased PGE2 level, inhibited β‐catenin signalling pathway of Ls174T cells with goblet cell phenotype^9^ and improved the differentiation of goblet cells. With regard to the effects of COX‐2/PGE2 on apoptosis, overexpression of COX‐2/PGE2 could promote apoptosis in several types of cells.^26^, ^27^, ^28^, ^29^, ^30^, ^31^ The impacts of COX‐2 inhibitors on apoptosis of cells in vitro are dependent on their doses. The high concentration of celecoxib (50‐100 mmol/L) may increase their apoptosis.^32^, ^33^, ^34^ Nevertheless, the low concentration of celecoxib in this study presented relatively less apoptosis because of proper inhibition of PGE2 in Ls174T cells (Figure S3).

Previous studies ever suggested the interaction between COX‐2/PGE2 and β‐catenin pathway. It has been reported that up‐regulating COX‐2 was associated with accumulation of β‐catenin and promoted the repair of intestinal mucosa after injury.^35^ We observed that the expression of pGSK3β was decreased by celecoxib but increased by PGE2, indicating COX‐2/PGE2 could control β‐catenin pathway by regulating the activity of GSK3β. The inflammation mediated by COX‐2/PGE2‐β‐catenin signalling pathway was characterized as low quality of intestinal epithelial proliferation due to their poor differentiation and excessive apoptosis. Targeting this pathway, celecoxib might be beneficial to alleviate liver cirrhosis via improvement of intestinal epithelial proliferation quality.

Actually, the intestinal inflammation associated with COX‐2 catalytic products in other digestive diseases present similar problems. The increased PGE2 could decrease barrier integrity via its receptor EP4 in patients with colitis.^36^ Another example is that during Entamoeba histolytica infection, the PGE2 produced by these pathogens increased ion permeability of tight junctions which led to diarrhoea.^37^ Moreover, the increase in PGE2 could modify intestinal microbiota, which also exacerbated intestinal inflammation.^38^ Colonic delivery of celecoxib ever showed the anti‐colitic efficiency in rats.^39^ Celecoxib, as a selective COX‐2 inhibitor, has been widely used for patients with osteoarthritis. It has presented good safety. This facilitates translation of the new treatment concept proposed in this paper towards clinical practice.

In conclusion, COX‐2/PGE2‐β‐catenin signalling pathway was involved in ileal epithelial inflammation in the rats with cirrhosis. The inflammation was characterized as low quality of intestinal epithelial proliferation due to their poor differentiation and excessive apoptosis. Targeting this pathway, celecoxib might be beneficial to alleviate liver cirrhosis via improvement of intestinal epithelial proliferation quality and ileal epithelial homeostasis.

## CONFLICT OF INTEREST

The authors confirm that there are no conflicts of interest.

## AUTHOR CONTRIBUTIONS

**Linhao Zhang:** Conceptualization (lead); Data curation (lead); Formal analysis (lead); Funding acquisition (equal); Investigation (lead); Validation (lead); Writing‐original draft (lead). **Yang Tai:** Funding acquisition (equal); Investigation (equal); Validation (equal). **Chong Zhao:** Formal analysis (equal); Investigation (equal); Methodology (equal). **Xiao Ma:** Data curation (supporting); Formal analysis (supporting); Investigation (equal); Methodology (supporting). **Shihang Tang:** Data curation (supporting); Formal analysis (supporting); Investigation (equal); Methodology (supporting). **Huan Tong:** Formal analysis (supporting); Methodology (supporting); Writing‐original draft (supporting). **Chengwei Tang:** Conceptualization (equal); Funding acquisition (lead); Methodology (supporting); Project administration (lead); Supervision (lead); Writing‐review & editing (lead). **Jinhang Gao:** Conceptualization (equal); Methodology (equal); Project administration (equal); Supervision (equal); Writing‐original draft (equal); Writing‐review & editing (equal).

## Supporting information

Supplementary Material

## Data Availability

The data that support the findings of this study are available from the corresponding author Jinhang Gao (jinhang@wchscu.cn or Gao.jinhang@qq.com) upon reasonable request.

## References

[1] Rath E, Moschetta A, Haller D. Mitochondrial function ‐ gatekeeper of intestinal epithelial cell homeostasis. Nat Rev Gastroenterol Hepatol. 2018;15:497‐516. 10.1038/s41575-018-0021-x 29844587

[2] Okumura R, Takeda K. Roles of intestinal epithelial cells in the maintenance of gut homeostasis. Exp Mol Med. 2017;49:e338. 10.1038/emm.2017.20 28546564 PMC5454438

[3] Negroni A, Cucchiara S, Stronati L. Apoptosis, necrosis, and necroptosis in the gut and intestinal homeostasis. Mediators Inflamm. 2015;2015:1‐10. 10.1155/2015/250762 PMC4592906 26483605

[4] Thoo L, Noti M, Krebs P. Keep calm: the intestinal barrier at the interface of peace and war. Cell Death Dis. 2019;10:849. 10.1038/s41419-019-2086-z 31699962 PMC6838056

[5] Pijls KE, Jonkers DM, Elamin EE, Masclee AA, Koek GH. Intestinal epithelial barrier function in liver cirrhosis: an extensive review of the literature. Liver Int. 2013;33:1457‐1469. 10.1111/liv.12271 23879434

[6] Wang Y, Zeng Z, Guan L, Ao R. GRHL2 induces liver fibrosis and intestinal mucosal barrier dysfunction in non‐alcoholic fatty liver disease via microRNA‐200 and the MAPK pathway. J Cell Mol Med. 2020;24:6107‐6119. 10.1111/jcmm.15212 32324317 PMC7294114

[7] Zhang L, Tai Y, Tang S, et al. Compromised ileal mucus barrier due to impaired epithelial homeostasis caused by notch1 signaling in cirrhotic rats. Dig Dis Sci. 2020;66(1):131‐142. 10.1007/s10620-020-06178-6 32144600

[8] Sorribas M, Jakob MO, Yilmaz B, et al. FXR modulates the gut‐vascular barrier by regulating the entry sites for bacterial translocation in experimental cirrhosis. J Hepatol. 2019;71:1126‐1140. 10.1016/j.jhep.2019.06.017 31295531

[9] Pope JL, Bhat AA, Sharma A, et al. Claudin‐1 regulates intestinal epithelial homeostasis through the modulation of Notch‐signalling. Gut. 2014;63:622‐634. 10.1136/gutjnl-2012-304241 23766441 PMC4083824

[10] Sehrawat TS, Liu M, Shah VH. The knowns and unknowns of treatment for alcoholic hepatitis. Lancet Gastroenterol Hepatol. 2020;5:494‐506. 10.1016/S2468-1253(19)30326-7 32277902 PMC7238289

[11] De Munck TJI, Xu P, Verwijs HJA, et al. Intestinal permeability in human nonalcoholic fatty liver disease: a systematic review and meta‐analysis. Liver Int. 2020;40(12):2906‐2916. 10.1111/liv.14696 33037768 PMC7756870

[12] Gao JH, Wen SL, Tong H, et al. Inhibition of cyclooxygenase‐2 alleviates liver cirrhosis via improvement of the dysfunctional gut‐liver axis in rats. Am J Physiol Gastrointest Liver Physiol. 2016;310(11):G962‐G972. 10.1152/ajpgi.00428.2015 27056726

[13] Li Y, Soendergaard C, Bergenheim FH, et al. COX‐2‐PGE2 signaling impairs intestinal epithelial regeneration and associates with TNF inhibitor responsiveness in ulcerative colitis. EBioMedicine. 2018;36:497‐507. 10.1016/j.ebiom.2018.08.040 30190207 PMC6197735

[14] Gao JH, Wen SL, Feng S, et al. Celecoxib and octreotide synergistically ameliorate portal hypertension via inhibition of angiogenesis in cirrhotic rats. Angiogenesis. 2016;19:501‐511. 10.1007/s10456-016-9522-9 27380212 PMC5026725

[15] Wen SL, Gao JH, Yang WJ, et al. Celecoxib attenuates hepatic cirrhosis through inhibition of epithelial‐to‐mesenchymal transition of hepatocytes. J Gastroenterol Hepatol. 2014;29:1932‐1942. 10.1111/jgh.12641 24909904

[16] Gao JH, Wen SL, Yang WJ, et al. Celecoxib ameliorates portal hypertension of the cirrhotic rats through the dual inhibitory effects on the intrahepatic fibrosis and angiogenesis. PLoS One. 2013;8:e69309. 10.1371/journal.pone.0069309 23922700 PMC3724827

[17] Moparthi L, Koch S. Wnt signaling in intestinal inflammation. Differentiation. 2019;108:24‐32. 10.1016/j.diff.2019.01.002 30718056

[18] Bae JS, Jeon Y, Kim SM, et al. Depletion of MOB1A/B causes intestinal epithelial degeneration by suppressing Wnt activity and activating BMP/TGF‐beta signaling. Cell Death Dis. 2018;9:1083. 10.1038/s41419-018-1138-0 30349003 PMC6197243

[19] Kriz V, Wnt KV. RSPO and hippo signalling in the intestine and intestinal stem cells. Genes. 2018;9(1):20. 10.3390/genes9010020 PMC5793173 29316729

[20] Schmitt M, Schewe M, Sacchetti A, et al. Paneth cells respond to inflammation and contribute to tissue regeneration by acquiring stem‐like features through SCF/c‐kit signaling. Cell Rep. 2018;24(9):2312‐2328.e7. 10.1016/j.celrep.2018.07.085 30157426

[21] Dudhwala ZM, Drew PA, Howarth GS, Moore D, Cummins AG. Active beta‐catenin signaling in the small intestine of humans during infancy. Dig Dis Sci. 2019;64:76‐83. 10.1007/s10620-018-5286-y 30382540

[22] Kretzschmar K, Clevers H. Wnt/beta‐catenin signaling in adult mammalian epithelial stem cells. Dev Biol. 2017;428:273‐282. 10.1016/j.ydbio.2017.05.015 28526587

[23] Zeng H, Ishaq SL, Zhao FQ, Wright AG. Colonic inflammation accompanies an increase of beta‐catenin signaling and Lachnospiraceae/Streptococcaceae bacteria in the hind gut of high‐fat diet‐fed mice. J Nutr Biochem. 2016;35:30‐36. 10.1016/j.jnutbio.2016.05.015 27362974

[24] Wang A, Li J, Zhao Y, Johansson ME, Xu H, Ghishan FK. Loss of NHE8 expression impairs intestinal mucosal integrity. Am J Physiol Gastrointest Liver Physiol. 2015;309(11):G855‐G864. 10.1152/ajpgi.00278.2015 26505975 PMC4669351

[25] Albillos A, de Gottardi A, Rescigno M. The gut‐liver axis in liver disease: Pathophysiological basis for therapy. J Hepatol. 2020;72:558‐577. 10.1016/j.jhep.2019.10.003 31622696

[26] Brea R, Motiño O, Francés D, et al. PGE2 induces apoptosis of hepatic stellate cells and attenuates liver fibrosis in mice by downregulating miR‐23a‐5p and miR‐28a‐5p. Biochim Biophys Acta Mol Basis Dis. 2018;1864:325‐337. 10.1016/j.bbadis.2017.11.001 29109031

[27] Guan PP, Liang YY, Cao LL, Yu X, Wang P. Cyclooxygenase‐2 induced the beta‐amyloid protein deposition and neuronal apoptosis via upregulating the synthesis of prostaglandin e2 and 15‐deoxy‐delta(12,14)‐prostaglandin J2. Neurotherapeutics. 2019;16:1255‐1268. 10.1007/s13311-019-00770-z 31392591 PMC6985346

[28] Martel‐Pelletier J, Pelletier JP, Fahmi H. Cyclooxygenase‐2 and prostaglandins in articular tissues. Semin Arthritis Rheum. 2003;33:155‐167. 10.1016/s0049-0172(03)00134-3 14671726

[29] Palumbo S, Toscano CD, Parente L, Weigert R, Bosetti F. The cyclooxygenase‐2 pathway via the PGE(2) EP2 receptor contributes to oligodendrocytes apoptosis in cuprizone‐induced demyelination. J Neurochem. 2012;121:418‐427. 10.1111/j.1471-4159.2011.07363.x 21699540 PMC3220805

[30] Xu Z, Choudhary S, Voznesensky O, et al. Overexpression of COX‐2 in human osteosarcoma cells decreases proliferation and increases apoptosis. Cancer Res. 2006;66:6657‐6664. 10.1158/0008-5472.CAN-05-3624 16818639

[31] Lan KC, Chiu CY, Kao CW, et al. Advanced glycation end‐products induce apoptosis in pancreatic islet endothelial cells via NF‐kappaB‐activated cyclooxygenase‐2/prostaglandin E2 up‐regulation. PLoS One. 2015;10:e0124418. 10.1371/journal.pone.0124418 25898207 PMC4405342

[32] Shi Z, Chen Y, Pei Y, et al. The role of cyclooxygenase‐2 in the protection against apoptosis in vascular endothelial cells induced by cigarette smoking. J Thorac Dis. 2017;9:30‐41. 10.21037/jtd.2017.01.23 28203404 PMC5303115

[33] Gee J, Lee IL, Jendiroba D, Fischer SM, Grossman HB, Sabichi AL. Selective cyclooxygenase‐2 inhibitors inhibit growth and induce apoptosis of bladder cancer. Oncol Rep. 2006;15:471‐477. 16391871

[34] Lev‐Ari S, Kazanov D, Liberman E, Ben‐Yosef R, Arber N. Down‐regulation of PGE2 by physiologic levels of celecoxib is not sufficient to induce apoptosis or inhibit cell proliferation in human colon carcinoma cell lines. Dig Dis Sci. 2007;52:1128‐1133. 10.1007/s10620-006-9619-x 17342386

[35] Lai Y, Zhong W, Yu T, et al. Rebamipide promotes the regeneration of aspirin‐induced small‐intestine mucosal injury through accumulation of beta‐catenin. PLoS One. 2015;10:e0132031. 10.1371/journal.pone.0132031 26135128 PMC4489841

[36] Lejeune M, Leung P, Beck PL, Chadee K. Role of EP4 receptor and prostaglandin transporter in prostaglandin E2‐induced alteration in colonic epithelial barrier integrity. Am J Physiol Gastrointest Liver Physiol. 2010;299:G1097‐G1105. 10.1152/ajpgi.00280.2010 20813914

[37] Lejeune M, Moreau F, Chadee K. Prostaglandin E2 produced by Entamoeba histolytica signals via EP4 receptor and alters claudin‐4 to increase ion permeability of tight junctions. Am J Pathol. 2011;179:807‐818. 10.1016/j.ajpath.2011.05.001 21683675 PMC3157226

[38] Crittenden S, Goepp M, Pollock J, et al. Prostaglandin E2 promotes intestinal inflammation via inhibiting microbiota‐dependent regulatory T cells. Sci Adv. 2021;7:eabd7954. 10.1126/sciadv.abd7954 PMC7880593 33579710

[39] Kim W, Lee Y, Jeong S, Nam J, Lee S, Jung Y. Colonic delivery of celecoxib is a potential pharmaceutical strategy for repositioning the selective COX‐2 inhibitor as an anti‐colitic agent. Arch Pharm Res. 2015;38:1830‐1838. 10.1007/s12272-015-0602-y 25860026

