# Supplementary material for: Inhibition of cyclooxygenase‐2 enhanced intestinal epithelial homeostasis via suppressing β‐catenin signalling pathway in experimental liver fibrosis
Source: J Cell Mol Med. 2021 Jun 19;25(16):7993–8005. doi: 10.1111/jcmm.16730 (PMC8358882; doi:10.1111/jcmm.16730)
Supplement: Supplementary file 1 — Supplementary Material [file JCMM-25-7993-s001.docx]

***Original Research***

**Inhibition of cyclooxygenase-2 enhanced intestinal epithelial homeostasis via suppressing β-catenin signaling pathway in experimental liver fibrosis**

Linhao Zhang^1, 2^, Yang Tai^1, 2^, Chong Zhao^1, 2^, Xiao Ma^1, 2^, Shihang Tang^1, 2^, Huan Tong^1, 2^, Chengwei Tang^1, 2, #^, Jinhang Gao^1, 2, #^

*^1^ Lab. of Gastroenterology & Hepatology, State Key Laboratory of Biotherapy, West China Hospital, Sichuan University, Chengdu 610041, China.*

*^2^ Department of Gastroenterology, West China Hospital, Sichuan University, Chengdu 610041, China.*

**^#^Correspondence authors:**

Chengwei Tang, MD, PhD, Professor

Department of Gastroenterology,

West China Hospital, Sichuan University

Guo Xue Lane 37#, Chengdu 610041, PR China

Tel.: 86-28-85422383

Fax: 86-28-85582944

E-mail: [shcqcdmed@163.com](mailto:shcqcdmed@163.com)

Jinhang Gao, M.D., PhD., Associate Professor

Lab of Gastroenterology and Hepatology,

West China Hospital, Sichuan University,

NO. 1, 4^th^ Keyuan Road, Chengdu, 610041, China.

Tel: 86-28-85164011

Fax: 86-28-85582944

E-mail: [Gao.jinhang@qq.com](mailto:Gao.jinhang@qq.com), jinhang@wchscu.cn

Supporting information


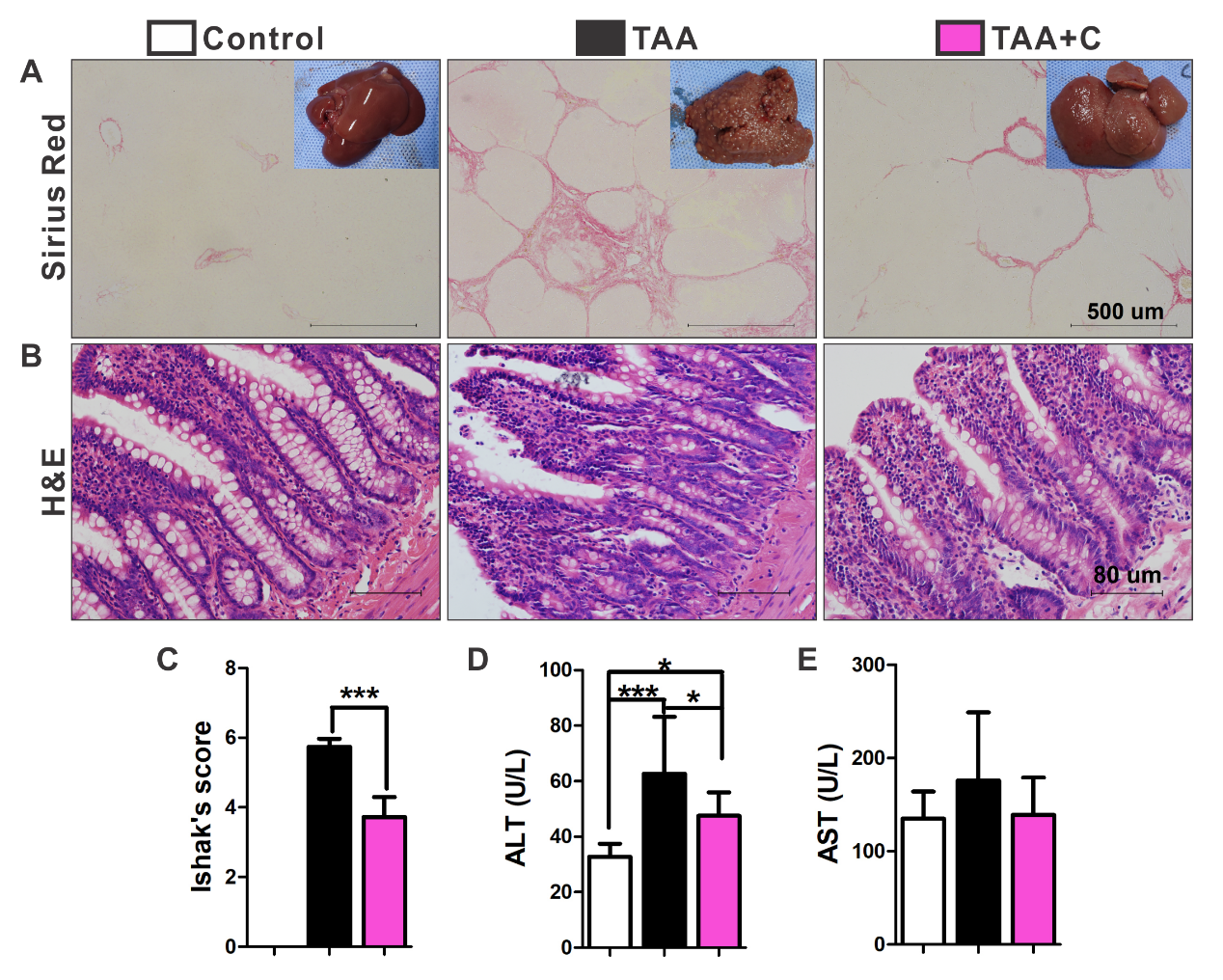


Supporting Figure 1

TAA group showed obvious liver fibrosis in gross and after staining with Sirius Red, which was significantly alleviated by celecoxib treatment (A, C). No difference could be identified by hematoxylin and eosin (H&E) staining in rat ileum (B). Serum alanine aminotransferase (ALT) was significantly higher, but the increase in aspartate aminotransferase (AST) was not significant in the TAA group, and these values were relatively lower in the TAA+C group (D-E, n=10-12 in each group). All experiments consisted of a minimum of 3 replicates. Data are shown as mean ± SD; * p<0.05, ** p<0.01, *** p<0.001; one-way ANOVA with Tukey’s post-hoc test.


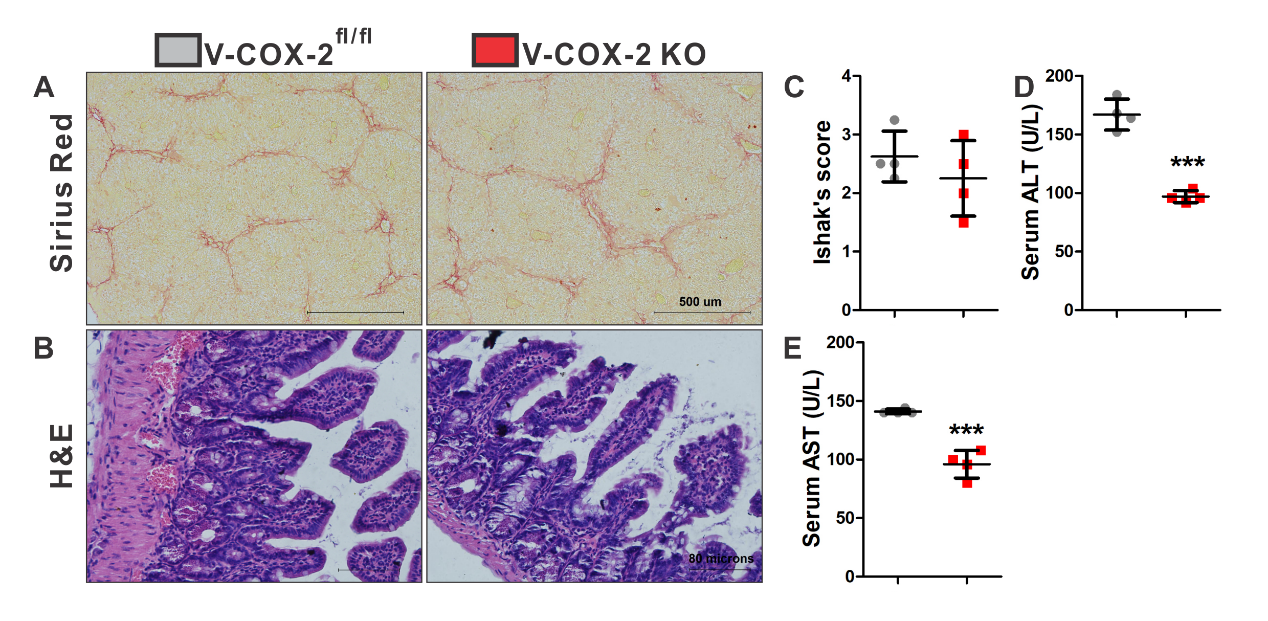


Supporting Figure 2

Liver fibrosis determined by Sirius Red stain was similar in mice (A, C). No difference could be identified by H&E staining in mice ileum (B). Serum ALT and AST were significantly lower in V-COX-2 KO mice (D-E). n=4 in each group in all experiments. All experiments consisted of a minimum of 3 replicates. Data are shown as mean ± SD; * p<0.05, ** p<0.01, *** p<0.001; two-tailed t-test.


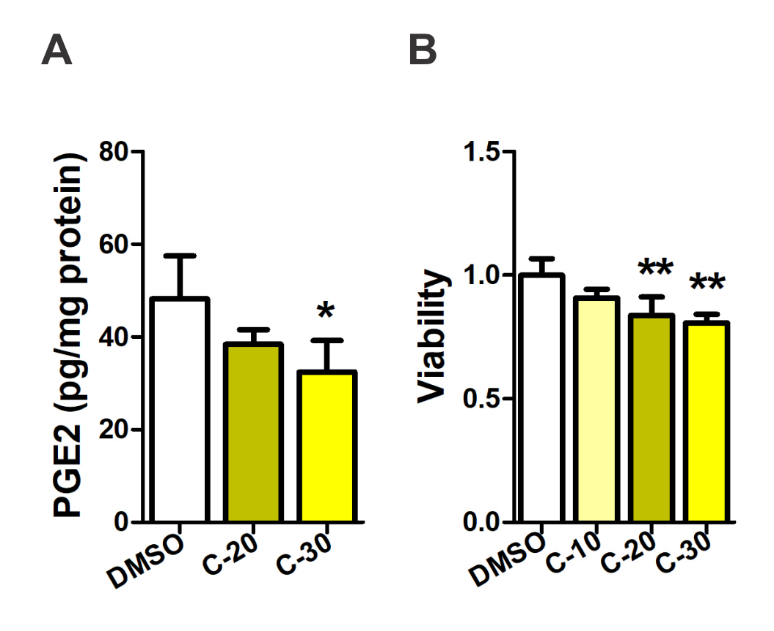


Supporting Figure 3

Both PGE2 concentration (A) and cell viability (B) could be reduced by 30 μM celecoxib.
